# Fathers’ perspectives on the breastfeeding experience: a qualitative study

**DOI:** 10.3389/fnut.2026.1813294

**Published:** 2026-05-19

**Authors:** Wiebke Stritter, Oana Costea, Theresa Philomena Ertlmaier, Anja Borgmann-Staudt, Monika Berns, Sarah B. Blakeslee, Magdalena Balcerek

**Affiliations:** 1 Charité – Universitätsmedizin Berlin, Department of Pediatric Oncology and Hematology, Berlin, Germany; 2 Charité – Universitätsmedizin Berlin, Charité Competence Center for Traditional and Integrative Medicine, Berlin, Germany; 3 Department of Gynecology and Obstetrics, Gemeinschaftskrankenhaus Havelhöhe, Berlin, Germany; 4 Charité – Universitätsmedizin Berlin, Department of Neonatology, Berlin, Germany; 5 Medical Department II – Oncology, Gastroenterology, Hepatology, Pneumology, Universitätsklinikum Leipzig, Leipzig, Germany

**Keywords:** breastfeeding, fathers, paternal involvement, infant feeding, qualitative research

## Abstract

Breastfeeding offers numerous short-, medium-, and long-term benefits for the physical and mental health of both mother and child and strengthens the bond between them. As the extent of these benefits is associated with breastfeeding duration, the WHO recommends exclusive breastfeeding for the first 6 months and continuation alongside complementary foods until at least the end of the second year of life. While studies suggest that fathers play a crucial role in breastfeeding success and duration, comparatively less attention has been paid to how fathers experience and negotiate their role in the breastfeeding process in everyday family life. This qualitative study interviewed 11 fathers with a semi-structured guideline 2 months after the birth of their first child for an in-depth understanding of fathers’ expectations, attitudes, and involvement in the breastfeeding process. Fathers were recruited on postnatal wards and selected for the interviews based on age, mode of birth, breastfeeding situation 2 months postpartum, and migration background, aiming to achieve a heterogeneous sample. Most fathers recognized the benefits of breastfeeding but also noted disadvantages, such as reduced father involvement. Formula feeding was recognized as acceptable, especially when breastfeeding was not possible, and was sometimes viewed as enabling greater paternal participation and a more equitable distribution of caregiving. Fathers critiqued the societal pressure on mothers to breastfeed and perceived emotional barriers that hindered their own involvement in the feeding process and the father-child relationship. Most fathers expressed a desire to have a say in decisions, but also believed that the mother should have the final word. These findings suggest that fathers’ support for breastfeeding is embedded in a complex relational process shaped by appreciation, ambivalence, and negotiated involvement. Specific educational programs that actively include fathers could enhance their support in the breastfeeding process and thereby positively influence breastfeeding success. Father-inclusive education and support strategies should therefore address not only knowledge gaps, but also the emotional and relational complexities of the paternal role in breastfeeding.

## Introduction

Breastfeeding offers numerous short-, medium- and long-term health benefits for both mothers and children, providing optimal nutrition during the first 6 months to 2 years (1). Exclusive breastfeeding is estimated to prevent approximately 823,000 child deaths and 22,000 maternal deaths globally every year (2). The World Health Organization (3) aims to increase exclusive breastfeeding rates in infants in their first 6 months to at least 50% by 2025 (4). In Germany, initial breastfeeding rates are high (72–97%), but drop within the first months postpartum (71.7% exclusively breastfed at 2 weeks, 55.8% by 4 months), despite increases in overall breastfeeding rates over the past two decades (5).

Fathers are increasingly recognized as key influencers of breastfeeding success and duration. Well-informed and involved fathers who hold a positive attitude towards breastfeeding contribute to improved breastfeeding outcomes (6–12). In the context of breastfeeding, paternal involvement may include emotional encouragement, practical support for the mother, participation in infant care and household tasks, involvement in feeding-related decision-making, and support in accessing health services (12–14). Previous research suggests that fathers often view breastfeeding positively and wish to be supportive, but may also feel uncertain, insufficiently informed, or excluded from the breastfeeding process (9, 10, 15, 16). Moreover, partner support appears to be multifaceted, encompassing knowledge, practical help, encouragement, emotional support, and responsiveness to maternal needs; some forms of support may, however, be experienced as unhelpful if they are not attuned to the mother’s situation (12, 13, 17, 18). While previous studies have examined fathers’ views, attitudes, and roles in relation to breastfeeding, including experiences of support, uncertainty, and exclusion (9, 13, 16, 19), comparatively less is known about how fathers experience and negotiate the relational tensions that breastfeeding may create in everyday family life, particularly with regard to asymmetries in bonding, caregiving, and involvement. This qualitative study therefore aimed to explore fathers’ expectations, attitudes, and involvement in the breastfeeding process.

## Methods

The qualitative study was part of a larger mixed-method project on the individual experience of breastfeeding in first time parents (InSti Study). While embedded in a larger mixed-method project, the present article reports only the qualitative interview data and was not designed as an integrated mixed-methods analysis. Parents were recruited on the postnatal wards in three different hospital settings: a university-teaching hospital, a certified baby-friendly hospital and an anthroposophic baby-friendly hospital. Mothers were invited to participate in a longitudinal survey assessment of their breastfeeding situation and experience shortly after birth and at 2, 6 and 12 months postpartum (20). Additionally, mothers and fathers were invited to participate in independent qualitative, semi-structured interviews at 2 months postpartum to gain in-depth insights into their perspectives and experience (21). This time-point was chosen to reduce participant burden directly after birth and to respect the intimacy of the families during this new and vulnerable time on the one hand and on the other hand as it can be assumed that the breastfeeding process has now reached a certain routine (22).

For fathers, the study explored their experience with their partner’s breastfeeding and perceived role in this process using a qualitative approach based on structuring content analysis (23). The present manuscript reports the qualitative component of the larger mixed-method InSti study as a standalone analysis and does not aim to integrate qualitative and quantitative findings within this paper. Given the exploratory aim of understanding fathers’ subjective experiences, expectations, and perceived roles, the study was informed by an interpretive qualitative approach and analyzed using structuring qualitative content analysis. The main researcher and interviewer (OC) was a female Master’s student of psychology trained in qualitative research methods. The research team comprised three senior physicians and specialists in pediatrics (MBa, AB, MBe), a senior expert in qualitative research and psychologist/psychotherapist (WS), a senior social scientist with expertise in qualitative research (SBB), and an assistant doctor in gynecology and obstetrics and MD student (TE). The team’s combined expertise in clinical practice, qualitative methodology, and psychological research supported rigorous data collection, analysis, and interpretation. The study was approved by the ethics committee (EA2/105/22) and registered in the central study registry (3000616) of Charité – Universitätsmedizin Berlin. All participants provided written informed consent, and data processing adhered to data protection regulations. The study was conducted and reported in accordance with the COREQ (Consolidated Criteria for Reporting Qualitative Research) guidelines (24).

### Sampling

Fathers were approached at postnatal wards of the three study sites within the first days after birth, between 11/2022 and 04/2023. Eligibility criteria included first-time fatherhood, both parents aged ≥18 years, admission of the mother and term-born infant to the postnatal ward without any relevant separation, sufficient German language skills and access to internet and telephone. A total of 166 fathers agreed to participate in an interview. We employed a purposive, maximum variation sampling strategy (25) to ensure a diverse sample aiming to capture a wide range of experiences and perspectives. Fathers were selected based on age at inclusion (<30; 30 to 39; >40 years), mode of birth (vaginal; caesarean section), breastfeeding status at 2 months (exclusive, partial, weaned, never breastfed) and migration background. This information was obtained from hospital records, documentation at recruitment, and questionnaires completed by mothers as part of the underlying mixed-method study (20). Participants were recruited until thematic saturation was reached (26). Among those contacted (via telephone and/or e-mail) 11/17 (64.7%) agreed to participate in the interview. No potential participants explicitly refused to take part; non-participation was due to lack of response to contact attempts via email or phone.

### Data collection and management

Interviews were conducted from 01 to 06/2023, nearly all were performed online via Cisco WebEx, as most fathers found it challenging to leave home for extended periods and preferred staying close to the baby. One interview was conducted in person and another by telephone due to the father’s specific circumstances. Each interview was one-on-one between the father and a consistent interviewer (OC). Fathers were advised to find a quiet, undisturbed space and silence their phones. However, not all interviews were completely undisturbed—for example, one father had his baby with him for a significant portion of the interview. The interview guide was developed based on key topics identified from literature, supplemented by study-specific aspects and refinement by the study team. Based on previous literature, the interview guide addressed fathers’ attitudes towards breastfeeding, paternal role perceptions, involvement in feeding-related decision-making, experiences of support or exclusion, and the relevance of infant feeding for family relationships (9, 12–16, 19). Topics explored included the experience of: *(I) breastfeeding initiation, (II) current feeding situation, (III) expectations before birth, (IV) preparation and resources, (V) involvement in decision-making, (VI) challenges (VII) perception of the father role, (VIII) advantages and disadvantages of breastfeeding vs. bottle-feeding, (IX) family experiences and environment.* In addition, open-ended follow-up prompts and probes (e.g., asking for concrete examples, feelings, perceived challenges, and clarification of statements) were used to deepen and specify participants’ accounts. Interviews were audio recorded using a Dictaphone OLYMPUS Digital Voice Recorder WS-831, with additional notes taken by the interviewer without identifying personal details.

### Data preparation and analysis

Interviews were transcribed verbatim using the semantic-content transcription system by Kuckartz et al. (27). Transcription and transcript checking were performed once the data collection had been completed. Transcriptions were automatically generated using the open-source tool Whisper/WhisperX (28) and refined by the interviewer (OC) in MAXQDA version 24 (29). MAXQDA 24 was also used to support coding, category development, and data management throughout the analysis. To ensure accuracy, a second team member (MBa) proofread all transcripts against the audio files. Interviews were conducted and transcribed in German, and coding was performed on the original German transcripts to preserve contextual meaning. Quotations presented in the manuscript were translated into English by the research team for publication purposes. Data were analyzed following the structuring content analysis approach of Kuckartz (30), including multiple reading and coding steps to immerse in the data. Initial main categories were deductively derived from the interview guide and reflected broad topical areas addressed in the interviews, such as expectations before birth, involvement in feeding-related decision-making, perceived father role, challenges, and attitudes towards breastfeeding and bottle-feeding. These categories served as an initial analytic framework. An initial coding framework was developed by OC based on the interview guide and repeated reading of the first transcripts. This preliminary codebook was discussed and refined within the research team during the coding process. Definitions and examples for categories and subcategories were documented and iteratively revised until the final coding structure was agreed upon. At the same time, the analysis was not limited to predefined topics: subcategories and thematic differentiations were developed inductively through open coding, repeated reading, and iterative comparison across transcripts. The final coding structure thus combined deductively informed topical categories with inductively developed thematic refinement.

Coding quality was ensured by consensual coding or intercoder agreement (OC, TE/MBa), disagreements were resolved through consultation of a third reviewer (MBa/TE). Throughout data collection and analysis, data were regularly discussed within the study team (OC, TE, MBa) and with experts on qualitative research methods (WS, SBB) to discuss interpretations and strengthen analytic rigor. Analytic decisions, revisions of the coding framework, and category definitions were documented throughout the analytic process in memos and versioned coding files, thereby providing an audit trail. Participants did not provide feedback on the findings.

## Results

After 11 interviews, no new themes emerged and thematic saturation was deemed sufficient. Interview duration ranged from 30 min to 1 h 29 min. The average age of interviewed fathers was 34 years, 30% had a migrant background. At the time of the interview, two fathers’ partners had weaned from breastfeeding, and three reported mixed feeding with formula. Mode of birth was balanced: six partners had vaginal births, five a caesarean section. One father in the sample had twins. Characteristics of fathers interviewed are provided in Table 1.

**Table 1:** Characteristics of fathers interviewed (*n* = 11).

Characteristics	Interviewed fathers		
	m	n	%
Average age of the father at the time of the interview	34.00		
Age of the father at the time of the interview			
< 30 Years		2	18.18
30–39 Years		7	63.64
> 40 Years		2	18.18
Missing Data		0	-
Migrant background			
Yes		3	30
No		7	70
Missing Data		1	-
Employed			
Temporarily excused (e.g., parental leave)		0	0
Not employed (including students)		2	20
Part-time or hourly employed		1	10
Full-time employed		7	70
In training/education		0	0
Unemployed		0	0
Missing Data		1	-
Birth mode			
Vaginal		3	27.27
Vaginal, instrumental		3	27.27
Scheduled cesarean section		2	18.18
Emergency cesarean section		0	0
Unplanned cesarean section		3	27.27
Missing Data		0	-
Multiple sibling births			
Yes		1	9.09
No		10	90.01
Missing Data		0	-
Gender of infants*			
Female		6	50.00
Male		6	50.00
Missing Data		0	-
Breastfeeding status at 2 months postpartum			
Exclusively breastfed.		6	54.55
Mixed feeding (breast milk and additional fluids / infant formula / complementary foods)		3	27.27
No longer breastfed.		2	18.18
Never breastfed.		0	0
Missing Data		0	-
Father’s attitude towards breastfeeding, as perceived by the mother			
Supports breastfeeding and finds it important		8	80
Has no opinion		1	10
Did not want me to breastfeed		0	0
I do not know		1	10
Missing Data		1	-
Total		11	100

*One mother gave birth to twins, therefore 11 fathers/mothers with 12 newborns. m, mean; n, number.

Five major themes emerged regarding the fathers’ perspectives on breastfeeding: (I) fathers’ attitudes towards breastfeeding, (II) attitudes towards formula feeding, (III) perception of the father-infant relationship, (IV) perceived social expectations of the maternal role and (V) situational breastfeeding attitudes (Supplementary material 1). Across themes, fathers’ perspectives were characterized by a fundamental appreciation of breastfeeding, combined with ambivalence about its consequences for paternal involvement, bonding, and family dynamics. While breastfeeding was often viewed as the optimal and emotionally meaningful form of infant feeding, fathers also described tensions related to passivity, unequal caregiving opportunities, and decision-making within the couple.

Within these broader themes, subthemes were developed inductively from the interview material, for example around fathers’ feelings of exclusion and passivity in the breastfeeding process, the perceived relevance of formula feeding for paternal involvement, and differing views within couples regarding breastfeeding-related decisions.

### Fathers’ attitudes towards breastfeeding

#### Perceived advantages

Fathers generally expressed a positive attitude and motivation towards breastfeeding, viewing it as the optimal, natural and healthiest choice for infant feeding. This belief was supported by health-related beliefs and personal accounts. Most fathers felt that breastfeeding’s advantages outweighed any disadvantages, some saw no disadvantage at all. Practical benefits, such as convenience and cost, were also appreciated to make everyday life easier, e.g., being easier than bottle feeding, free and they did not need to worry about the feeding.

Breastfeeding also held emotional meaning for many fathers. Some described it as an emotional experience that strengthens the child’s comfort, maternal well-being and the mother-infant bond, e.g., by skin-to-skin contact and intimacy, which helps calm the baby and creates a protective space.

*It’s also a huge protective space for the little one[….*] *I know, complementary food is great and all that. But it’s SKIN CONTACT. […]* *(F3).*

One father reported that breastfeeding motivated him to support his partner more actively and seek higher involvement in the child’s everyday life. In his account, this seemed to be related to appreciation of the mother’s efforts and a desire to contribute more actively to the family’s everyday care. Another described breastfeeding as a source of inspiration, even prompting him to sing and feel more connected.

#### Perceived disadvantages

Several disadvantages of breastfeeding were noted; however, only by a few fathers. Especially the inevitable inequality between parents created by breastfeeding with the child primarily depending on the mother was emphasized. This led to some fathers feeling less able to support and needing to find other ways to bond.

*I’m mainly seen by him while changing nappies and she[‘s seen] while breastfeeding.*

*And swaddling usually leads […] to him crying […]. And when he cries, the solution is to breastfeed.*

*In* other *words, […So,] SHE is almost always the one who gets him out of a situation where he has to cry and I, or the fact that I’m swaddling him, leads to him starting to cry (F9).*

Fathers regretted their limited role in the breastfeeding process and that they were unable to breastfeed themselves, which they thought led to passivity on their part. They noted that emotional connections were paramount, even over health benefits. Additionally, some mentioned the disadvantage of changes to the mother’s body, the feeling that the breast now belonged to the child, and the uncertainty around the child’s milk intake. Generally, fathers prioritized a stress-free feeding experience and strong emotional bonds for the entire family over any specific feeding method.

*[Y]es, of course, breast milk is the best nourishment for the baby, but if it stresses you out, [.]* then *it’s no good, right? And then it’s better that you find an alternative, right? (F5).*

For some fathers, breastfeeding thus represented not only a valued feeding practice, but also a relational arrangement that could heighten their sense of passivity and unequal involvement.

### Father’s attitudes towards formula feeding

#### Perceived advantages

Some fathers compared formula favorably to breast milk, while others felt it could not replicate its benefits. Most stated that feeding decisions, including whether to choose breast milk or formula, were individual choices for the couple, and each decision could be based on good reasons. Some fathers acknowledged formula as a useful alternative when breastfeeding was not possible or desired. Though breast milk was widely considered the optimal nutrition form, some fathers stated that this does not automatically imply formula being harmful. Some believed that formula had a good reputation and assumed it nutritionally well adapted to the child’s needs, similar to breast milk.

*I think* formula *is an absolutely great thing. It works.*

*Babies* grow *from it and it’s absolutely necessary to have for people who REALLY, REALLY need it.*

*That is* important *(F3).*

Fathers appreciated the practicality of formula, including being able to track the child’s intake. Many saw it as a way to engage more actively in caregiving, fostering equity with the parental relationship with their child. For some formula was a relief that enabled more physical closeness and bonding, while creating a safe space for the baby. Simultaneously, formula feeding allowed mothers more personal time and to be less dependent on the child.

*[B]ottle-feeding makes a certain amount of equality possible.*

*In care work […] I think that’s the big advantage for me (F1).*

Formula was therefore described not only as an alternative nutritional option, but also as a way of increasing paternal participation and partially reducing the asymmetry associated with breastfeeding.

#### Perceived disadvantages

Despite acknowledging some benefits, many fathers felt formula lacked key advantages of breastmilk, such as nutrients specifically composed to the child’s needs for healthy growth and immune system support.

*And with bottle feeding [formula] it’s also partly the same as with adults. We eat what we have in our fridge [. With formula y]ou do not have the antibodies against infectious pathogens that are currently flooding our home and environment. […] And the mother shares this system [protection] with the baby (F10).*

Some were skeptical of formula’s production and safety, stating potential economic marketing motives. Further concerns related to bottle-feeding were raised, including potential sucking confusion, doubts about its soothing effect, and a possible loss of intimacy compared to breastfeeding. Some fathers admitted that they lacked knowledge about formula.

### Perception of the father-infant relationship

#### Perceived influence of breastfeeding

Breastfeeding was seen to initially strengthen the mother-infant bond more than the father-infant bond, assuming that as a result the infant might develop different associations to its parents. This led to feelings of exclusion, less involvement and passivity in some fathers. They noted that they could not soothe the infant when hungry and only feeling recognized by the infant during tasks like diaper changes. To compensate, some fathers sought alternative bonding activities such as holding, bathing and swaddling, helping them bond with their child and take a more active role as a father. While some acknowledged breastfeeding naturally strengthening the mother-infant relationship, they did not see it as diminishing their role as father.

*So it might be that the relationship between the baby and mother becomes closer, but I do not think that would make the relationship between the father and baby any less close (F7).*

Some even regretted not having an own breastfeeding experience but accepted it, understanding that breastfeeding was ultimately best for their child. Fathers reported a range of emotions while observing their baby being breastfed, from neutrality to feelings of happiness, fulfillment and satisfaction. Some described it as a moment of family unity, marked by a calm yet connected atmosphere, rich with shared presence.

*You can definitely say, [the role breastfeeding plays in my relationship with my baby is] a big one (laughing). […] I know that everything is going well and I know that she feels comfortable there, [and] that I can totally rely on my wife, for example. She does an amazing job, always making the little one feel good.*

*And I can recharge myself, so to speak at the same time. So like I see it, it gives me strength. […]So it builds it up, […t]he relationship between me and her too (F3).*

*[S]ometimes [I] wish for a bit more [of a relationship between the baby and me if she wasn’t being breastfed] […] I would feed her more often then, […] Yeah, so [breastfeeding] would just have a little less magic and maybe a little more magic on my side (F3).*

For some fathers, breastfeeding thus not only shaped feeding practices, but also created a relational asymmetry in early caregiving and bonding, which could be experienced as emotionally challenging.

#### Perceived influence of bottle-feeding

The fathers whose partners had stopped breastfeeding said bonding was harder while their child was still breastfed, as they felt limited to a passive observer. When fathers, whose partners were still breastfeeding, were asked to imagine the father-infant relationship without breastfeeding, some believed it would be closer, others saw no difference, and a few were uncertain. Fathers who bottle-fed their baby, regardless of the current breastfeeding status, generally felt it positively influenced their relationship with the child, strengthening bonding and trust. Feeding helped them to recognize hunger cues and respond adequately, which provided relief and an empowering sense of involvement. Some described bottle-feeding as creating a safe space for the baby. Some fathers particularly enjoyed feeding the baby in the carrier which contributed to a stronger closeness. Interestingly, some fathers referred to bottle-feeding as “breastfeeding.”

*Yes, definitely. I do think that [bottle feeding] promotes bonding and also feeding with fingers was bond-promoting back then. […] I was sort of the substitute mom, if you will, right? (F5).*

These accounts suggest that bottle-feeding could, for some fathers, compensate for the limited involvement they had experienced during breastfeeding.

### Perceived social expectations of the maternal role

#### Societal pressure on mother to breastfeed

Few fathers recognized that breastfeeding is an emotionally charged topic in society. They criticized the lack of a genuine choice for women, as social expectations – including from family and friends – contribute to a strong expectation for mothers to breastfeed. Some mothers were reported to transfer this pressure on themselves. Some fathers expressed their empathy for women who could not or chose not to breastfeed and emphasized that acceptance of parents breastfeeding choices should be the norm within families and society. Fathers stated that the child’s feeding should be adapted to the respective situation, e.g., if the mother did not want to or could not breastfeed.

*Yes, it’s definitely not easy because overall it’s an emotionally charged topic in society.*

*It puts women, especially my wife, under additional pressure. I do not know if that’s really helpful (F8).*

*In today’s— in our society, it’s not really a choice for women, which I find almost shocking. I also find it shocking that for a time it wasn’t a choice—that bottle feeding was given in all cases and breastfeeding was somehow frowned upon. But at the same time, I find it difficult right now that there’s no room for flexibility. It’s just assumed. And, I find it creates significant moral pressure on women and young mothers. And if this pressure exists and it’s expected that mothers breastfeed, then they should breastfeed of course. But then they should get the proper support so that it’s possible for every woman without difficulty (F8).*

#### Challenges in breastfeeding

Factors mentioned to complicate breastfeeding included maternal health issues, physical discomfort or lactation problems, which could result in stress or frustration, as well as factors related to the child, such as preference for bottles.

#### Maternal motivation to breastfeed

Fathers shared insights into their partners’ motivation regarding breastfeeding. Many fathers perceived that for the mothers breastfeeding was important, with some of them loving breastfeeding and looking forward to breastfeeding their child again after several hours. Some fathers noted that the mother had wanted to approach breastfeeding more flexibly, with an open mindset, wanting to try it out without pressure. One father mentioned his partner’s mixed feelings; at times not wanting to continue breastfeeding, only to later consider that she might want to continue for an extended period. Another father described having had to motivate his partner to breastfeed for 6 weeks before they mutually decided to wean and transition to bottle-feeding.

#### Body

Fathers generally viewed post-pregnancy physical changes associated with breastfeeding as natural and not problematic if one chose to have a child. With the mother breastfeeding, fathers believed, that her breasts were now primarily “dedicated to the child”. Some also noted that breastfeeding had negative esthetic effects on the mother’s breasts, though they saw these as minor disadvantages compared to the benefits of breastfeeding.

*From a male perspective you could say that, of course a woman’s breasts are naturally affected from breastfeeding. But that is rather a very small disadvantage. And […] only […] for the man […] (F7).*

One father said that his partner did not believe breastfeeding necessarily caused noticeable changes and he shared that it was not a concern for him either.

#### Admiration of the mother

Many fathers were proud of how their partner managed to successfully breastfeed from the very beginning. They expressed deep admiration for their partners and described them as wonderful mothers who they could rely on and trust. Fathers felt that the mother represented the most beautiful aspect of their baby’s world.

*And I am very, very proud of my wife because she puts so much time and energy into it, especially at the beginning, when it did not work. […] My wife kept with it and showed an great deal of ambition (laughing) so that it eventually worked at some point (F7).*

#### Attitudes towards breastfeeding in public

Most fathers affirmed that breastfeeding in public should be considered as normal and expressed no personal objections to their partner or other women publicly breastfeeding. However, they felt that their home offered a more comfortable and intimate environment for breastfeeding. They expressed concerns that their partners might feel stared at or judged while publicly nursing, worrying about reactions of others. Some admitted feeling uncomfortable themselves when another woman breastfed in their presence as they were unsure where to direct their gaze at that moment.

*I always found it a little bit difficult […] as a man, you can only go wrong somehow. If you stare, it might be bad. (Inhales) If you deliberately do not look, then you are somehow prudish, I do not know. […] Otherwise, uh, I think private breastfeeding is probably nicer, because it’s more quiet, but of course there are a lot of occasions where it is necessary to do it publicly, and then it definitely should not be a problem (F6).*

### Situational breastfeeding attitudes

#### Presumption of breastfeeding before birth

Many fathers presumed before the birth of their child, that breastfeeding would be an easy, instinctive and natural process believing the child to latch and feed without difficulties. In hindsight, they found it more challenging than anticipated. At the same time, some had feared breastfeeding might fail, citing concerns like milk stasis, pain, nipple problems and low milk production. Others had worried that aids, such as bottles or shields, could lead to sucking confusion or dependence. One father doubted that exclusive breastfeeding was possible with twins. Generally, fathers believed breastfeeding would be less time-consuming than bottle-feeding. Despite these concerns, many couples had hoped to breastfeed successfully. Some fathers had a basic trust that—with time and practice—breastfeeding would become easier, even if challenges arose.

*Before the [childbirth] course I simply thought, that’s natural. Basically, you put the baby to the breast and it drinks. I never thought about the possibility that there might be problems with it. [During our birth preparation course….] two films were shown […], where it was shown how mothers breastfeed their children and what is important […] I think that was the first time I thought that it might not be so easy or that there might be problems. […] I would not have thought beforehand that there would be difficulties with us, because it’s like such a natural process (F6).*

#### Role models

Fathers’ personal histories regarding breastfeeding were also likely to play a role in their attitudes and decisions. Most of the fathers knew that they themselves had been breastfed, while some fathers were uncertain. Some fathers recalled reports from their mothers about having struggled with breastfeeding or that they had found weaning stressful. Fathers also had additional personal exposure to breastfeeding, e.g., through their own siblings, giving them a sense of being familiar with breastfeeding, including sharpening of the understanding of its challenges and rewards.

#### Participation in the decision-making processes regarding breastfeeding

Most fathers emphasized the importance of involvement in breastfeeding decisions while recognizing that their partners should have the final say, as it directly affects their bodies and experiences.

*Yes, it is definitely pretty important to me to at least be informed. [, but in the end…] it’s somehow HER decision and all, but (inhales) nevertheless, I think that, as I said, I also have a lot of experience that could, for example simply lead to a slightly different outcome if I brought that into play (F3).*

Some felt that they had limited decision-making power, believing that the decision was entirely up to the mother and child, yet still desired to be consulted. Only a few fathers were indifferent. One father explicitly stated involvement in breastfeeding decisions not being important to him but cared about decisions regarding formula and preferred to focus on other parenting aspects, such as practicing tummy time with his child.

Differing opinions on breastfeeding sometimes led to conflicts in some couples with some fathers stating occasionally different ideas about how certain things should be handled. For instance, one father wished to freeze excess milk, which his partner found inconvenient; another felt that a sleeping child should not be woken to breastfeed. In many families, there was no prior discussion about whether to breastfeed or not; breastfeeding was taken for granted. Among those who had discussed it, all agreed to breastfeed.

*So I definitely had some influence, uh, because as I also often told her: she should consider whether it still makes sense, because uh because it makes her so unhappy.*

*(Inhales) […] It was also important to me that I do not just impose or force it on her […].*

*In the end it’s her decision and yeah, I definitely held back because I also knew that it was simply so important to her to breastfeed the baby. Exactly (F11).*

Although some fathers described differing opinions or conflicts regarding breastfeeding-related decisions, most also emphasized that the mother should have the final say because breastfeeding directly affected her body and experience. The data therefore suggest limits to paternal influence in some situations, but do not indicate a uniform pattern of fathers being actively excluded from decision-making by mothers.

#### Breastfeeding duration

Opinions on breastfeeding duration varied. Many fathers stated not having thought about it in detail yet, while others suggested milestones like the mother’s return to work, daycare or the child’s reduced need for breast milk as weaning points. Only a few supported extended breastfeeding, leaving the decision up to the mother and child. Contradictions also surfaced; for example, one father left the decision to his partner, but privately opposed very short (2 months) or very long (2 years) durations. Two fathers were comfortable with their child being breastfed for 2 years. Most fathers, however, were not this specific.

#### Motivation for weaning

The reasons for (potential) weaning varied, with some fathers highlighting the emotional strain on their partners. Further reasons included breastfeeding difficulties such as hospital-related challenges, medication use and side effects, trouble with positioning the baby and insufficient milk production.

*Somehow my wife was so EXHAUSTED, shall we say, that she […] at some point was no longer willing to breastfeed or to try to stimulate milk flow so that there would be enough milk (F5).*

Among the two fathers whose partners had already weaned, attitudes differed. One initially encouraged continued breastfeeding but changed advising towards weaning when his partner remained unhappy with breastfeeding, which had led to him also feeling unhappy and sad. For the other, weaning brought relief after severe stress and emotional exhaustion associated with breastfeeding. He reported subtly urging weaning, though he stated that at other points he had withdrawn from the decision to wean to give his partner the space to decide.

*I kept kind of insisting that my wife should just wean because she was just extremely unhappy And the baby was also just so unhappy and it always led to really huge stressful situations and that of course also made ME unhappy. Or sad too (F11).*

Both fathers ultimately supported weaning, recognizing the stress breastfeeding had placed on their partners. In hindsight, both were content and relieved, viewing the decision to wean as beneficial for family dynamics.

## Discussion

This qualitative study provides first-hand insights into fathers’ perceptions of breastfeeding—an often underrepresented aspect in research (15). Through in-depth interviews, we comprehensively explored fathers’ breastfeeding perceptions and experiences.

Before birth, most fathers anticipated breastfeeding to be a simple, natural process. In practice, however, they encountered challenges, with breastfeeding difficulties being commonly underestimated by both parents (31). This discrepancy highlights the value of preparing fathers specifically for difficulties, equipping them with coping strategies and realistic expectations to enable empathetic support during breastfeeding stress. Despite this mismatch, most fathers overall remained motivated for their child to be breastfed. The majority revealed a positive attitude towards breastfeeding, consistent with previous studies (9, 10, 15, 16), showing that fathers often value breastfeeding and see it as beneficial for child health often grounded on personal experiences or scientific findings. Beyond this, our findings suggest that such positive attitudes were often embedded in broader emotional and relational meanings, including admiration for the mother and a desire to support family well-being. Paternal attitude plays a pivotal role in breastfeeding outcomes: fathers confident in their ability to support breastfeeding are more likely to be actively involved, which increases initiation and duration (6, 9, 10, 12). Most fathers expressed a differentiated view towards breastfeeding versus formula—reflecting realistic, well-informed perspectives. Only in individual cases did they not see a difference. Various studies emphasize that paternal knowledge is a central component of breastfeeding support (6, 10, 12, 13). Most fathers correctly identified breastfeeding benefits for the child’s health, however only one addressed maternal health benefits, without specifying them. This suggests prenatal education should more explicitly address maternal advantages to promote a more comprehensive understanding.

Another key theme was breastfeeding’s impact on the father-infant relationship. Fathers expressed mixed feelings about the impact of breastfeeding on the father–infant relationship, echoing previous reports of paternal exclusion and frustration (9, 15, 19). Extending this literature, our findings show that fathers did not simply feel excluded. Many actively sought alternative ways of creating closeness and involvement, suggesting that paternal engagement in the breastfeeding context is negotiated rather than absent. Fathers recognized breastfeeding creating a unique often more intense bond between mother and child than their own. This perception may even result in feelings of envy (19). Some fathers described breastfeeding as creating asymmetries in bonding, caregiving, and decision-making. However, our data more clearly suggest situational limits to paternal involvement than a consistent pattern of maternal exclusion.

Active participating in feeding, as by bottle feeding or introduction of solids, has been described as critical to paternal engagement (19). While previous studies have highlighted fathers’ involvement in infant feeding and the importance of active participation (12, 19), our findings add that formula feeding was sometimes viewed by fathers not only as a practical or nutritional alternative, but as a way of partially rebalancing caregiving and strengthening paternal participation. In our study, many fathers reported how they engaged in alternative activities for creating strong relationships, like changing diapers, carrying or bedtime routines. These are also important activities aligning with Sherriffs et al. (12) five-dimensional frameworks of paternal support: attitude, knowledge, participation, emotional and practical support, including shielding the mother from external stressors, navigating health services, and fostering a positive breastfeeding environment (14). Several studies show that emotional, physical and social support from fathers strengthens the mother’s self-efficacy and likelihood of breastfeeding success (7, 32, 33).

Most fathers expressed a strong interest in participating in breastfeeding and weaning decisions, affirming that the final choice belonged to the mother. Their expectations for breastfeeding duration ranged from a few months to up to 2 years or as long as possible. Some fathers linked breastfeeding duration to practical family transitions, such as the mother’s return to work or the child’s start in daycare. Many acknowledged that it was the mother’s decision of when to stop breastfeeding, given the physical and emotional demands, whereas others emphasized the child’s readiness would be a trigger for weaning for them. Consistent with Dutheil et al. (34), the mother’s return to work was a frequently cited reason for weaning as well as breastfeeding difficulties, maternal dissatisfaction and emotional stress. In this context, several believed that discontinuing breastfeeding was appropriate in some contexts and criticized social pressure placed on mothers and the lack of freedom of choice, echoing findings of Flothkötter et al. (35). Simultaneously, many expressed admirations of the mother’s breastfeeding achievements.

In two contrasting cases, fathers either encouraged continuation or subtly promoted weaning, illustrating the delicate balance of involvement. Fathers involved in decision-making and knowledgeable about breastfeeding are better able to support their partners and help overcome breastfeeding difficulties more successfully (12). Targeted paternal involvement can even double the likelihood of a baby being exclusively breastfed for 6 months (8). Yet, support must remain responsive to the mother’s needs: Previous literature suggests that over-involvement, such as unsolicited advice or persistent encouragement, can inadvertently apply pressure and undermine maternal autonomy contributing to early weaning (36). In our study, a few fathers described actively encouraging either continuation of breastfeeding or weaning, indicating that paternal involvement could at times become emotionally delicate. However, over-involvement did not emerge as a consistent theme across the interviews. Even well-intentioned encouragement can cause stress and earlier weaning (17, 18). This highlights the emotional complexity of paternal involvement suggesting that future interventions should inform fathers not only about breastfeeding but also prepare them for emotional dynamics it may generate, including effective support strategies.

Many fathers supported public breastfeeding and considered normalization desirable. However, they also expressed a certain ambivalence. Some felt uneasy about others judging their partners. Further, own insecurities when women breastfeed in their presence were addressed. Such shame and discomfort have also previously been reported (16). This internal conflict suggests the persistent need of cultural and societal sensitization to foster acceptance, in which breastfeeding is seen as normal and unremarkable. Adequate public promotion could further reduce stigma, empower mothers and strengthen a supportive breastfeeding environment.

### Limitations

Several limitations must be acknowledged. While efforts were made to support reflexivity and analytic quality through regular team discussions, interviewer influence cannot be excluded, particularly as the interviewer was a breastfeeding mother during data collection. This positionality may have shaped both the interview dynamics and the fathers’ willingness to express critical or ambivalent views on breastfeeding. In addition, interviews were coded only after data collection had been completed, while earlier coding might have allowed adaptations to the interview guide for emerging topics.

A further limitation concerns the sample and the available sociodemographic data. No fathers were interviewed whose partners had never breastfed, and same-sex partners were not included. Although variation in age was sought, few younger or older fathers participated. Moreover, father-specific socioeconomic data were limited, as the questionnaire component was completed by mothers. While some broader family-level contextual information was available, socioeconomic indicators such as fathers’ education and income were not systematically assessed for the fathers themselves. This limits our ability to reflect on how socioeconomic circumstances may have shaped fathers’ perspectives, opportunities for involvement, and support needs. Similarly, migration-related information was limited and did not allow an in-depth exploration of cultural influences on fathers’ breastfeeding perspectives.

Self-selection bias must also be considered, as participation may have been more feasible or attractive for fathers who were particularly motivated, reflective, or engaged in the breastfeeding process. Fathers who felt less involved, less informed, or more critical of breastfeeding may therefore be underrepresented. Finally, only interview segments relevant to the research question were analyzed, material not examined in depth may have contained further insights beyond the scope of the present study. Future research should examine fathers’ experiences across more diverse social and family contexts and further explore fathers’ specific needs, particularly around fostering responsive breastfeeding support without generating additional pressure.

## Conclusion

This study aimed to explore fathers’ experiences, perspectives, and perceived role in the breastfeeding process. Beyond prior work showing that fathers are associated with breastfeeding outcomes, our study adds a first-hand account of how fathers themselves experience breastfeeding as a valued yet sometimes asymmetrical process that shapes bonding, caregiving, and involvement within the family. Fathers in our study largely supported breastfeeding, but their accounts show that such support was often accompanied by feelings of asymmetry, passivity, and negotiated involvement, highlighting breastfeeding as not only a feeding practice but also a relational family process. Fathers are therefore crucial yet often overlooked partners in breastfeeding. When supported, informed, and emotionally prepared, they can contribute significantly to successful breastfeeding outcomes and family well-being. Our findings point to a need for father-inclusive education and support strategies that address not only knowledge gaps, but also the emotional, relational, and social complexities of the paternal role. To maximize the health benefits of breastfeeding, future initiatives should focus on integrating fathers more meaningfully into breastfeeding support structures.

## Data Availability

The data supporting the conclusions of this article are available from the corresponding author on reasonable request, subject to ethical and data protection restrictions.

## References

[1] Miolski J. Benefits of breastfeeding for mother and child. Sanamed. (2023) 18:59–63. doi: 10.5937/sanamed0-41390

[2] Prentice AM. Breastfeeding in the modern world. Ann Nutr Metab. (2022) 78:29–38. doi: 10.1159/000524354 35679837

[3] Asimaki E Dagla M Sarantaki A Iliadou M. Main biopsychosocial factors influencing breastfeeding: a systematic review. Maedica (Bucur). (2022) 17:955–62. doi: 10.26574/maedica.2022.17.4.955, 36818247 PMC9923068

[4] WHO. WHO Response. Infant and young child Feeding 2023 (2026); Available online at: https://www.who.int/news-room/fact-sheets/detail/infant-and-young-child-feeding#:~:text=WHO%20response,infant%20and%20young%20child%20feeding. (Accessed February 16, 2026).

[5] Hockamp N Burak C Sievers E Rudloff S Burmann A Thinnes M . Breast-feeding promotion in hospitals and prospective breast-feeding rates during the first year of life in two national surveys 1997-1998 and 2017-2019 in Germany. Public Health Nutr. (2021) 24:2411–23. doi: 10.1017/S1368980021001099, 33722333 PMC10195543

[6] Atkinson L Silverio SA Bick D Fallon V. Relationships between paternal attitudes, paternal involvement, and infant-feeding outcomes: mixed-methods findings from a global on-line survey of English-speaking fathers. Matern Child Nutr. (2021) 17:e13147. doi: 10.1111/mcn.13147 34241959 PMC8269144

[7] Abbass-Dick J Brown HK Jackson KT Rempel L Dennis CL. Perinatal breastfeeding interventions including fathers/partners: a systematic review of the literature. Midwifery. (2019) 75:41–51. doi: 10.1016/j.midw.2019.04.001, 30999255

[8] Mahesh PKB Gunathunga MW Arnold SM Jayasinghe C Pathirana S Makarim MF . Effectiveness of targeting fathers for breastfeeding promotion: systematic review and meta-analysis. BMC Public Health. (2018) 18:1140. doi: 10.1186/s12889-018-6037-x, 30249216 PMC6154400

[9] Earle S Hadley R. Men's views and experiences of infant feeding: a qualitative systematic review. Matern Child Nutr. (2018) 14:e12586. doi: 10.1111/mcn.12586, 29349895 PMC6866241

[10] Hansen E Tesch L Ayton J. They're born to get breastfed'- how fathers view breastfeeding: a mixed method study. BMC Pregnancy Childbirth. (2018) 18:238. doi: 10.1186/s12884-018-1827-9, 29914401 PMC6006837

[11] Tadesse K Zelenko O Mulugeta A Gallegos D. Effectiveness of breastfeeding interventions delivered to fathers in low- and middle-income countries: a systematic review. Matern Child Nutr. (2018) 14:e12612. doi: 10.1111/mcn.12612, 29740958 PMC6866104

[12] Sherriff N Hall V Panton C. Engaging and supporting fathers to promote breast feeding: a concept analysis. Midwifery. (2014) 30:667–77. doi: 10.1016/j.midw.2013.07.014, 23958385

[13] Davidson EL Ollerton RL. Partner behaviours improving breastfeeding outcomes: an integrative review. Women Birth. (2020) 33:e15–23. doi: 10.1016/j.wombi.2019.05.010 31196832

[14] Mgolozeli S. Perceived roles of fathers in the promotion, support and protection of breastfeeding. Africa J Nurs Midwifery. (2018) 20:1–19. doi: 10.25159/2520-5293/4060

[15] Sihota H Oliffe J Kelly MT McCuaig F. Fathers' experiences and perspectives of breastfeeding: a scoping review. Am J Mens Health. (2019) 13:1557988319851616. doi: 10.1177/1557988319851616, 31092114 PMC6537273

[16] Merritt R Vogel M Ladbury P Johnson S. A qualitative study to explore fathers' attitudes towards breastfeeding in south West England. Prim Health Care Res Dev. (2019) 20:e24. doi: 10.1017/S1463423618000877, 32799968 PMC6476394

[17] Emmott EH Mace R. Practical support from fathers and grandmothers is associated with lower levels of breastfeeding in the UK millennium cohort study. PLoS One. (2015) 10:e0133547. doi: 10.1371/journal.pone.0133547, 26192993 PMC4507871

[18] Rempel LA Rempel JK Moore KCJ. Relationships between types of father breastfeeding support and breastfeeding outcomes. Matern Child Nutr. (2017) 13:e12337. doi: 10.1111/mcn.12337, 27460557 PMC6865933

[19] de Montigny F Larivière-Bastien D Gervais C St-Arneault K Dubeau D Devault A. Fathers’ perspectives on their relationship with their infant in the context of breastfeeding. J Fam Issues. (2018) 39:478–502. doi: 10.1177/0192513X16650922

[20] Ertlmaier TP Costea O Borgmann-Staudt A Berns M Weikert G Kersting M . How can we support the individual breastfeeding experience? Quantitative results from a mixed-methods study. Int Breastfeed J. (2025) 20:38. doi: 10.1186/s13006-025-00726-4, 40382664 PMC12085814

[21] Johnson JM. "In-Depth Interviewing". In: Gubrium JF Holstein JA, editors. Handbook of Interview Research: Context and Method. Thousand Oaks: Sage Publications (2022). p. 106–24.

[22] Both D Frischknecht K. Stillen kompakt - Atlas zur Diagnostik und Therapie in der Stillberatung, vol. 1 München: Elsevier GmbH (2007).

[23] Kuckartz U. "Qualitative text analysis: a systematic approach". In: Kaiser G Presmeg N, editors. Compendium for Early Career Researchers in Mathematics Education. Cham: Springer International Publishing (2019). p. 181–97.

[24] Tong A Sainsbury P Craig J. Consolidated criteria for reporting qualitative research (COREQ): a 32-item checklist for interviews and focus groups. Int J Qual Health Care. (2007) 19:349–57. doi: 10.1093/intqhc/mzm042, 17872937

[25] Schreier M. "Sampling and generalization". In: The SAGE Handbook of Qualitative Data Collection. London: SAGE Publications Ltd (2018). p. 84–97.

[26] Saunders B Sim J Kingstone T Baker S Waterfield J Bartlam B . Saturation in qualitative research: exploring its conceptualization and operationalization. Qual Quant. (2018) 52:1893–907. doi: 10.1007/s11135-017-0574-8, 29937585 PMC5993836

[27] Kuckartz U. Qualitative Evaluation: Der Einstieg in die Praxis, vol. 2. Wiesbaden: VS Verlag für Sozialwissenschaften (2008).

[28] Bain M. WhisperX: Time-Accurate Speech Transcription of Long-Form Audio. Dublin, Ireland: INTERSPEECH (2023).

[29] VERBI Software. MAXQDA [Computer Software]. (2024); Available online at: https://www.maxqda.com. (Accessed February 16, 2026).

[30] Kuckartz U. Qualitative Inhaltsanalyse. Methoden, Praxis, Computerunterstützung, vol. 4 Weinheim, Basel: Beltz Juventa in der Verlagsgruppe Beltz (2018).

[31] Tucker Z O'Malley C. Mental health benefits of breastfeeding: a literature review. Cureus. (2022) 14:e29199. doi: 10.7759/cureus.29199, 36258949 PMC9572809

[32] Uludağ E Öztürk S. The effect of partner support on self-efficiency in breastfeeding in the early postpartum period. Am J Fam Ther. (2020) 48:211–9. doi: 10.1080/01926187.2019.1697973

[33] Purwaningsih H Azizah N Rosalina R Mintarsih S. How can support from fathers increase breastfeeding in children? KnE Life Sci. (2022), 427–434. doi: 10.18502/kls.v7i2.10337

[34] Dutheil F Méchin G Vorilhon P Benson AC Bottet A Clinchamps M . Breastfeeding after returning to work: a systematic review and Meta-analysis. Int J Environ Res Public Health. (2021) 18:631. doi: 10.3390/ijerph18168631, 34444380 PMC8393856

[35] Flothkötter M Kunath J Lücke S Reiss K Menzel J Weikert C. Becoming breastfeeding friendly in Germany-an international research project to assess the readiness to scale up breastfeeding. Bundesgesundheitsbl Gesundheitsforsch Gesundheitsschutz. (2018) 61:1012–21. doi: 10.1007/s00103-018-2784-1, 30014189

[36] Modak A Ronghe V Gomase KP. The psychological benefits of breastfeeding: fostering maternal well-being and child development. Cureus. (2023) 15:e46730. doi: 10.7759/cureus.46730, 38021634 PMC10631302

