# Supplementary material for: Fathers’ perspectives on the breastfeeding experience: a qualitative study
Source: Front Nutr. 2026 May 19;13:1813294. doi: 10.3389/fnut.2026.1813294 (PMC13226198; doi:10.3389/fnut.2026.1813294)
Supplement: Supplementary file 1 [file Table_1.docx]

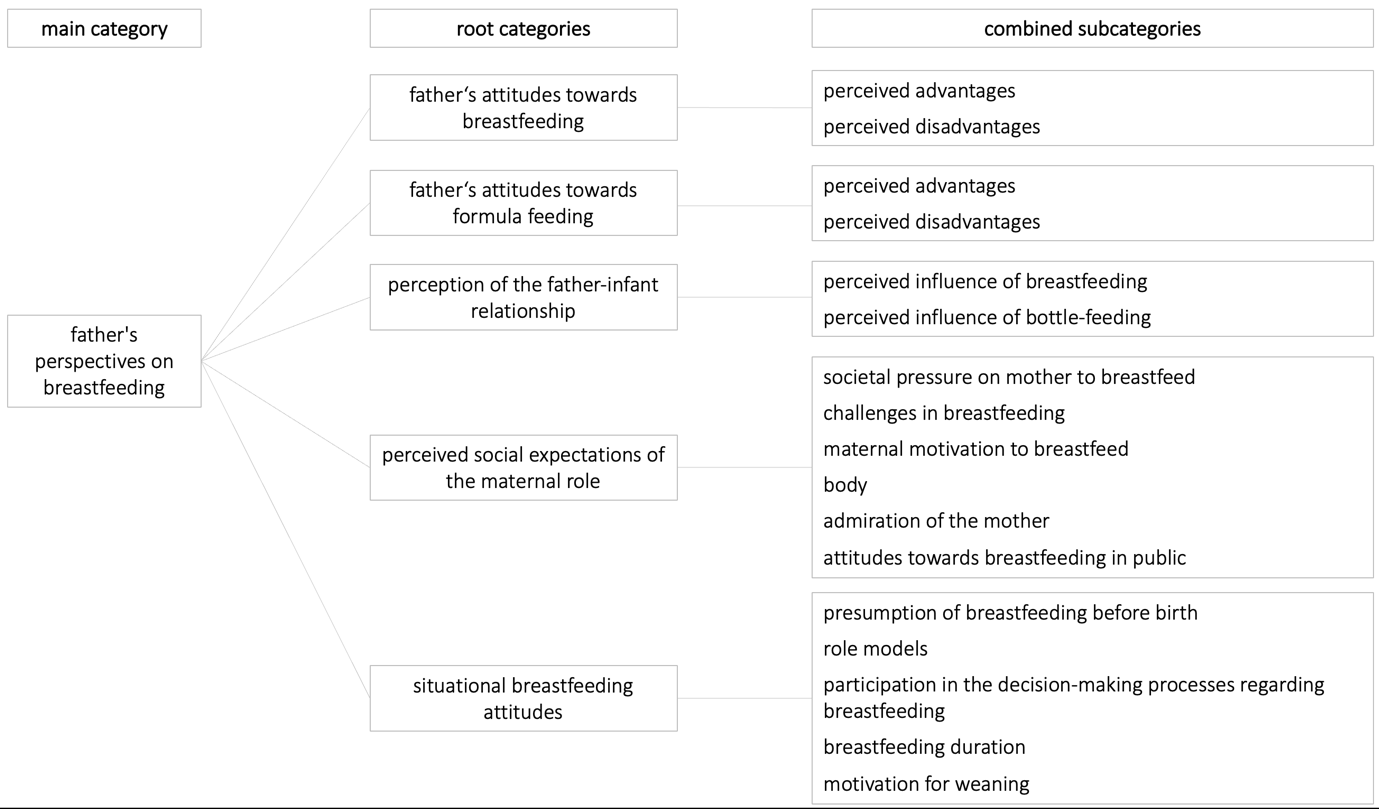


Supplement 1: Overview of main categories and subcategories on the topic father's perspectives on breastfeeding
